# Editorial for the Topic on Advanced Laser Fabrication Technologies for Cross-Field Applications

**DOI:** 10.3390/mi15091138

**Published:** 2024-09-07

**Authors:** Zhuo-Chen Ma, Xue-Qing Liu, Bing Han

**Affiliations:** 1 Institute of Medical Robotics, School of Biomedical Engineering, Shanghai Jiao Tong University, Shanghai 200240, China; zcma@sjtu.edu.cn; 2 Department of Automation, Shanghai Jiao Tong University, Shanghai 200240, China; 3 State Key Laboratory of Robotics and Systems, Harbin Institute of Technology, Harbin 150001, China; 4 State Key Laboratory of Integrated Optoelectronics, College of Electronic Science and Engineering, Jilin University, Changchun 130012, China; liuxueqing@jlu.edu.cn

The field of laser fabrication technologies has seen remarkable advancements in recent years. Over the past few decades, lasers have evolved from basic light sources into highly sophisticated tools [1,2], typified by ultrafast femtosecond lasers that enable unparalleled precision in manufacturing [3,4,5]. These developments have opened the door to a wide range of applications, from the precise cutting and engraving of materials to the fabrication of complex micro-/nanostructures with exceptional accuracy [6,7,8]. In this Editorial on advanced laser fabrication technologies for cross-field applications, we summarize 14 cutting-edge papers that highlight the diversity of applications and innovations within the field of laser fabrication technologies. These contributions can be broadly categorized into four key areas: (1) the optimization of laser fabrication techniques, (2) the processing of diverse materials, (3) the fabrication of 3D micro-/nanostructures, and (4) customized applications.

Lasers have become indispensable in material processing due to their ability to deliver energy with high precision, enabling localized heating, melting, and ablation without affecting surrounding areas. In this Topic, three articles specifically focus on optimizing laser processing methods from various perspectives. Zhu et al. explore the use of Python scripting for the secondary development of Abaqus [9], aiming to enhance the simulation efficiency of laser shock forming processes. This approach is particularly significant for the rapid shaping of complex aluminum alloy plates used in high-speed trains. Focusing on fine-tuning laser parameters, their study provides a detailed analysis of deformation characteristics, leading to more precise control over the forming process and improved manufacturing efficiency. Zhang et al. employ a design of experiments (DOE) methodology to predict and control outcomes of femtosecond laser micromilling on quartz [10]. By examining the effects of various laser parameters on depth and surface roughness, they develop a predictive model that minimizes the need for extensive experimental trials, thereby reducing time and cost requirements. The model’s accuracy is confirmed through the successful fabrication of blind holes. Additionally, Jedrkiewicz et al. carried out laser beam shape optimization using ultrashort Bessel beams to drill through hard and brittle sapphire [11]. This approach achieves tailorable elongated microstructures with minimal surface damage and low taper angles, essential for high-precision applications. Combining Bessel beam machining with a trepanning technique further enhances the quality and efficiency of the drilling process. Collectively, these studies underscore the importance of optimizing laser fabrication techniques through advanced modeling, simulation, and innovative hybrid approaches.

To fully harness the immense potential of laser fabrication, it is essential to consider the materials being processed. This Topic includes four articles that delve into the laser-based processing of widely used industrial materials such as alumina ceramics, steel, and titanium dioxide. These studies not only explore the interaction between lasers and these materials but also optimize processing parameters to enhance material properties. For instance, Zhao et al. investigate the effects of laser power, scanning speed, and pulse frequency on the surface roughness of alumina ceramics [12]. Through a combination of orthogonal tests and the development of a transient two-dimensional model using COMSOL Multiphysics, they identify optimal polishing parameters. This study also provides valuable insights into the material flow dynamics within the molten pool during the polishing process, with the simulation results closely aligning with experimental data, thereby confirming the model’s accuracy and reliability. In another study, Zhao et al. also introduce an innovative approach for improving surface roughness in SKD61 die steel by integrating a steady magnetic field into the laser polishing process [13]. This hybrid technique significantly reduces surface roughness by effectively suppressing the secondary overflow of the molten pool. Furthermore, the development of a multi-physical field numerical transient model enhances the understanding of the interplay between heat transfer, laminar flow, and electromagnetic fields, demonstrating the potential of combining different physical fields to optimize laser-based material processing. Liu et al. present a hybrid femtosecond laser processing technique aimed at enhancing the photocatalytic performance of titanium dioxide [14]. By incorporating silver nanoparticles into the TiO2 structure, they achieve a significant improvement in catalytic efficiency, particularly in the photodegradation of organic pollutants. This novel approach overcomes the limitations of traditional TiO2 photocatalysts, such as their limited light absorption and inefficient charge carrier dynamics, making it a promising solution for environmental and energy-related challenges. Finally, the exploration of femtosecond laser-induced phase transformation in single-crystal 6H-SiC represents a major advancement in understanding the ablation mechanisms at the microscopic level [15]. Detailed analysis of phase transformation thresholds and structural modifications under varying energy inputs offers crucial insights into optimizing laser parameters for high-quality micromachining of hard materials like SiC, which is essential for the development of SiC-based MEMS devices. These studies collectively underscore the critical role of material considerations in laser fabrication, showcasing how the optimization of laser parameters and the integration of novel techniques can lead to significant advancements in industrial applications, particularly in sectors related to sensors [16], actuators [17,18], electronics [19], and micro-devices [20,21].

Beyond optimizing laser fabrication techniques for various materials, the ability to create micro- and nanostructures through laser-based technologies has led to significant breakthroughs in the fields of photonics, optics, and electronics. These advances are particularly evident in the creation of anti-reflective surfaces, micro-lens arrays, and photonic crystals, which are essential components in modern optical devices. One of the prominent techniques in this area is femtosecond direct laser writing (fs-DLW), which has demonstrated unique advantages in 3D micro- and nanostructuring [22]. For example, fs-DLW is used to fabricate microlens arrays (MLAs) and graphene oxide gratings with exceptional precision [23]. These micro-optical elements play a critical role in compact integrated optical systems. The work carried out by Hou et al. exemplifies how fs-DLW can overcome challenges in optical chip integration [24]. Their novel waveguide structure, designed to mitigate issues such as signal crosstalk and crossover, introduces photonic jumper wires and an optical overpass. This innovative approach facilitates more complex on-chip optical connections, advancing the potential for high-density optical interconnects and pushing the boundaries of photonic chip integration. In another groundbreaking study, Ma et al. employ a biomimetic approach to develop dynamic adhesion interfaces [25], inspired by the gecko’s ability to control adhesion strength. Using two-photon polymerization, they fabricate mushroom-shaped micropillars on a flexible PDMS substrate. These structures exhibit remarkable resilience and tunable adhesion properties, making them highly suitable for applications in microfluidics, flexible electronics, and micro-assembly. This research highlights the potential of laser-based fabrication in developing advanced materials with adaptable physical properties. Sui et al. explore biomimicry in laser fabrication by modifying aluminum alloy surfaces based on natural non-smooth textures [26,27]. By adjusting the spacing of reticulate units during laser cladding, they achieve notable improvements in microstructure, microhardness, and tensile properties. Their study identifies an optimal spacing unit of 2.5 mm, which provides the best combination of strength and toughness. This work offers valuable insights into the design of biomimetic surfaces to enhance material performance, particularly in applications where material resilience and toughness are critical. These examples provide readers with a deeper understanding of how laser-based fabrication technologies are driving advancements in material manipulation at the micro- and nanoscale.

The development of laser manufacturing technologies has brought tremendous convenience to both production and daily life, permeating a wide range of industries. In this Topic, we focus on two primary applications of laser manufacturing technologies. First, Ma et al. provide a comprehensive overview of the transformative role of femtosecond lasers (FSLs) in ophthalmic surgery [28]. From refractive surgery to cataract removal, FSL technology has revolutionized surgical procedures by offering unparalleled precision, safety, and efficacy. Their review also explores potential future developments in this field, underscoring the ongoing impact of laser technologies in improving clinical outcomes and expanding the capabilities of ophthalmic surgery. Additionally, Wang et al. address the challenges of creating anti-reflection surfaces on hard materials [29], which are crucial for applications in infrared imaging, optoelectronics, and aerospace. Their study summarizes recent advances in the field, highlighting the broad-spectrum anti-reflection properties achieved through femtosecond laser processing. It also discusses certain limitations and future directions for improving the performance and applicability of anti-reflection surfaces, emphasizing the importance of continued research in this area.

The articles mentioned in this Editorial collectively represent the vanguard of research on laser fabrication technologies. They not only demonstrate significant advancements in process optimization and material structuring but also highlight the wide-ranging applications of these technologies in fields such as environmental catalysis, micro-optics, precision surgery, and beyond. The insights and innovations presented in this collection may offer new solutions to both long-standing and emerging challenges related to the underlying science of advanced lasers, as well as their engineering and fabrication.
